# Risk and Protective Factors of Psychological Distress in Patients Who Recovered From COVID-19: The Role of Cognitive Reserve

**DOI:** 10.3389/fpsyg.2022.852218

**Published:** 2022-06-03

**Authors:** Maria Devita, Elisa Di Rosa, Pamela Iannizzi, Sara Bianconi, Sara Anastasia Contin, Simona Tiriolo, Marta Ghisi, Rossana Schiavo, Nicol Bernardinello, Elisabetta Cocconcelli, Elisabetta Balestro, Anna Maria Cattelan, Davide Leoni, Biancarosa Volpe, Daniela Mapelli

**Affiliations:** ^1^Department of General Psychology, University of Padua, Padua, Italy; ^2^Unitá Operativa Complessa (UOC) Hospital Psychology, Padua University Hospital, Padua, Italy; ^3^Department of Cardiac, Thoracic, Vascular Sciences and Public Health, University of Padua, Padua, Italy; ^4^Infectious Disease Unit, Padua University Hospital, Padua, Italy

**Keywords:** COVID-19, cognitive reserve, psychological distress, subjective cognitive difficulties, protective factors, risk factors

## Abstract

Recent studies reported the development of psychological distress symptoms in patients who recovered from COVID-19. However, evidence is still scarce and new data are needed to define the exact risk and protective factors that can explain the variability in symptoms manifestation. In this study, we enrolled 257 patients who recovered from COVID-19 and we evaluated the levels of psychological distress through the Symptoms Checklist-90-R scale. Data concerning illness-related variables were collected from medical records, while the presence of subjective cognitive difficulties, both before and after the illness, as well as the level of the cognitive reserve (CR), were assessed over a clinical interview. Results revealed that being female and reporting the presence of subjective cognitive difficulties after COVID-19 were associated with higher levels of psychological distress. At the same time, being admitted to the hospital and having a high CR were protective factors. Adding new information to this emerging research field, our results highlight the importance of a complete psychological and cognitive assessment in patients with COVID-19.

## Introduction

In the last year and a half, the entire world has gone through the outbreak of a novel coronavirus, the severe acute respiratory syndrome coronavirus 2 (SARS-CoV-2). So far, more than 200 million cases of infection have been detected worldwide and more than 4 million deaths have unfortunately been recorded (World Health Organization). Indeed, the severity of COVID-19, the disease caused by this infection, widely ranges. It can either be asymptomatic or, in its mild to moderate form, characterized by physical symptoms such as fever, cough, fatigue, and breathing difficulties. Neurological symptoms can also be present, such as loss of smell and taste, febrile seizures, convulsions, change in mental status, and encephalitis. In the most severe cases, COVID-19 can lead to a multisystemic disease often requiring oxygen therapy, hospitalization, and, in the worse cases, intensive care and intubation. It is now well-established that the main risk factors that increase physical symptoms’ severity are age, the presence of respiratory and cardiovascular diseases, and comorbidities, such as obesity, diabetes, and hypertension (Jordan et al., 2020; Matsushita et al., 2020; Passamonti et al., 2020; Sattar et al., 2020; Wolff et al., 2020; Zheng et al., 2020).

Importantly, symptoms of psychological distress, Anxiety, and post-traumatic stress have been reported among patients with COVID-19 (Xu et al., 2020; Bonazza et al., 2020; Janiri et al., 2020; Ma et al., 2020; Rabinovitz et al., 2020; Liyanage-Don et al., 2021), as well as months after recovery (Tomasoni et al., 2021). However, despite their impact on patients’ quality of life, the psychological symptoms of COVID-19 have been scarcely investigated if compared with the physical ones, with no available evidence about their risk and potential protective factors. Indeed, obtaining this information would be extremely useful to maximize both prevention and treatment strategies of the so-called long-COVID syndrome, and the persistence of physical and psychological symptoms in patients who recovered from COVID-19.

Hence, to overcome this limitation by adding new knowledge to this research field, in this study, we evaluated the levels of psychological distress in a large group of patients who recovered from COVID-19, aiming to reach the following three objectives.

The first one concerned the study of the relation between COVID-19 severity and psychological distress. Indeed, although previous evidence has been reported so far, the currently available studies have obtained mixed results (see Mazza et al., 2020; Liyanage-Don et al., 2021; Putri et al., 2021; Sykes et al., 2021; Vincent et al., 2021), and new evidence is needed to shed light on the role of illness-related experiences, such as hospitalization or the need of oxygen supply therapy and psychological distress symptoms of patients who recovered from COVID-19.

Second, we aimed at studying the existence of an association between psychological distress and subjective cognitive difficulties in patients who recovered from COVID-19.

In fact, despite the presence of illness-related cognitive failures has been widely documented (Ferrucci et al., 2021; Kumar et al., 2021; Miskowiak et al., 2021), as well as the association between cognitive frailties and subsequent mood disorders, to the best of our knowledge, scarce evidence these variables among patients with COVID-19 is available.

Concerning these first two objectives, we hypothesized that higher levels of psychological distress can be detected in those who experienced a higher level of COVID-19 severity and in those who reported the presence of cognitive failures.

Finally, our third objective was to evaluate the potential role of cognitive reserve (CR) in the development of psychological distress symptoms in patients with COVID-19. CR is the ability to compensate for brain damage through neural networks and alternative cognitive strategies (Stern, 2009). CR represents a sort of cumulative cerebral potential derived from everyday life (i.e., education, working, and leisure time activities that are cognitively engaging and stimulating). It was found to play protective effects against medical diseases (Ribeiro et al., 2020), although evidence for its influence on psychopathological conditions is still scarce. In the context of the COVID-19 pandemic, in which mood disorders were expected and reported by the current literature, it seems important to understand which factors may protect individuals from developing psychopathological issues (Coin et al., 2022). CR is supposed to have this role, thanks to the higher resilience (Ran et al., 2020), cognitive strategies, and resources people with high CR show every day. Hence, our hypothesis was to find a negative association between CR levels and psychological distress symptoms of COVID-19.

## Materials and Methods

### Participants

Two hundred and fifty-seven (110 F; mean age 57.3 ± 13.4 years; range 18–86 years; mean education 13.2 ± 4.7 years; range 5–30 years) participants were consecutively recruited, while accessing the follow-up clinical visit at the Infectious and Tropical Diseases Unit (Azienda Ospedale-Università Padova), within 1 month after hospital discharge, between May 2020 and June 2021. During a single follow-up, participants underwent neurological, ophthalmological, blood, cognitive, and psychological examinations. Inclusion criteria were previous positivity to syndrome coronavirus 2 (SARS-CoV-2), a previous consultation and/or hospitalization, and aged between 18 and 90 years. Exclusion criteria were being non-Italian native speakers and the presence of important physical/sensory deficits, which could not allow the testing sessions to properly take place. Moreover, patients with medical records indicating a history of psychiatric illness were also excluded from the analysis, as well as those participants who asked for the medical investigation because of their subjective cognitive impairments in the last year. Every participant provided written informed consent before entering the study. Data were collected at the hospital’s Psychology, the Infectious Diseases, and the Pneumology Operational Units of the Padova University Hospital, during post-hospitalization pneumatological and infectious follow-ups, occurring 1 month after the last negative nasopharyngeal swab test. The protocol was in accordance with the Helsinki Declaration on human rights and was approved by the Ethics Committee of the Padova University Hospital.

### Measures

Every participant was asked to attend a testing session where psychological distress was assessed through the *Symptoms Checklist-90-R* (SCL-90-R; Derogatis, 1992), which produces three Global Indices (Global Severity Index, Positive Symptom Distress Index, and Positive Symptom Total), as well as nine primary symptom dimensions (Somatization, Obsessive–Compulsive, Interpersonal Sensitivity, Depression, Anxiety, Hostility, Phobic Anxiety, Paranoid Ideation, and Psychoticism). Raw scores were transformed into *T* scores for all the 12 indices and, according to the normative values (Prunas et al., 2012), scores over 55 were considered clinically significant, with the range between 55 and 64 considered moderate severity and scores over 64 considered as major severity indices.

The presence of subjective cognitive difficulties (i.e., forgetfulness, concentration drop, attentional lability, and distractibility) before, during the last year, and after COVID-19 infection was assessed using a semi-structured, “*ad hoc*” clinical interviews. Answers have been categorized as binary variables (presence; yes vs. absence; no).

To evaluate CR, the *Cognitive Reserve Index Questionnaire* (CRIq; Nucci et al., 2012) was employed. Responses to this questionnaire produce one global index (CRIq total score), and three sub-scores reflecting the role of education (CRIq-education), occupation (CRIq-working activities), and stimulating activities not related to education and work (CRIq-leisure time).

Information about the severity of COVID-19 diseases, such as hospitalization, intubation,^1^ and oxygen supply,^2^ as well as the presence of neurological and previous psychiatric symptoms, was obtained from medical records.

### Statistical Analyses

Descriptive statistics were performed for each of the measure obtained. Chi-square tests were performed to investigate the association between the presence of subjective cognitive difficulties before and after COVID-19. Multiple linear regression models were conducted separately for each of the 12 indices of the SCL-90-R (*T* scores), which were considered dependent variables. To corroborate this methodological approach, a power analysis has been also conducted (λ = 38.55, *F* = 1.79, df = 12, 1 − β = 0.99). In each of the models, the following independent variables were considered: age, sex, the presence of subjective cognitive difficulties before COVID-19 (yes vs. no); the presence of subjective cognitive difficulties after COVID-19 (yes vs. no); the four CRIq scores (CRIq total score, CRIq-education, CRIq-working activities, and CRIq-leisure time), presence of neurological symptoms (yes vs. no), hospitalization (yes vs. no), intubation (yes vs. no), and oxygen therapy (yes vs. no).

Statistical analyses were carried out using JAMOVI software, version 1.6.

## Results

Data collected from the medical records indicate that 84% of the patients’ sample (*N* = 216) were hospitalized because of COVID-19, with 18.3% (*N* = 47) receiving an oxygen supply therapy (such as high flow nasal cannula, non-invasive ventilation, and intensive oxygen therapy) and 6.2% (*N* = 16) needing intubation. Of the total sample included, 60% of the patients (*N* = 156) experienced neurological symptoms of COVID-19, such as anosmia, ageusia, headache, olfactory and auditory hallucinations, and tinnitus.

Data collected in the clinical interview revealed that 32.3% of the patients (*N* = 83) reported the presence of cognitive difficulties after COVID-19, as shown in Table 1. Importantly, 75.9% of them (*N* = 63) declared that these difficulties were not present before COVID-19. Results of a Chi-square test confirmed the absence of a significant association between the presence of cognitive difficulties before and after COVID-19 (*x*^2^ = 0.248; *p* = 0.619).

**TABLE 1:** Shows demographic, COVID-19, and psychological descriptive characteristics of the sample, and the frequencies, among participants, of presence/absence of cognitive difficulties before and after infection.

	Descriptives	
	Mean	SD
Age	57.34	13.39
Education	13.22	4.71
CRIq CRI-education CRI-work CRI-leisure time **Cognitive Reserve Index Total Score (CRI-tot)**	105.70 109.41 123.85 116.78	14.99 18.58 19.28 18.30
GSI	48.77	11.94
PST	48.65	11.60
PSDI	47.44	10.33
SOM	50.83	11.92
O-C	49.33	12.44
I-S	46.72	10.15
DEP	50.40	12.23
ANX	49.96	11.60
HOS	45.39	7.83
PHOB	52.42	13.09
PAR	45.37	9.42
PSY	49.07	11.50
ISLEVEL	0.07	0.25

	**Subjective cognitive difficulties after COVID-19?**		
**Subjective cognitive difficulties before COVID-19?**	**Yes**	**No**	**Total**

Yes	20	47	67
No	63	127	190
Total	83	174	257

As it showed in Table 2, more than 20% of the sample showed clinically relevant scores at the SCL-90-R subscales assessing symptoms of Anxiety (22.6%), Depression (23%), Obsessive–Compulsive behavior (25.3%), Somatization (27.6%), and Phobic Anxiety (28.8%).

**TABLE 2:** Symptoms Checklist-90-R and Cognitive Reserve Index Questionnaire scores.

Symptoms Checklist-90-R	*T* score Mean (SD)	Clinically relevant (*T* ≥ 55) % (*N*)	Moderate (*T* ≥ 55-64) % (*N*)	Severe (*T* ≥ 65) % (*N*)
Global Severity Index	48.7 (11.9)	20.6 (53)	10.9 (28)	9.7 (25)
Positive Symptom Distress Index	47.4 (10.3)	21.4 (55)	14.8 (38)	6.6 (17)
Positive Symptom Total	48.6 (11.6)	26.8 (69)	13.6 (35)	13.2 (34)
Somatization	50.8 (11.9)	27.6 (71)	14.4 (37)	13.2 (34)
Obsessive–Compulsive	49.3 (12.4)	25.3 (65)	14.4 (37)	10.9 (28)
Interpersonal Sensitivity	46.7 (10.1)	14 (36)	7.4 (19)	6.6 (17)
Depression	50.4 (12.2)	23 (60)	13.2 (34)	10.1 (26)
Anxiety	49.9 (11.6)	22.6 (58)	13.2 (34)	9.3 (24)
Hostility	45.4 (7.8)	10.9 (28)	7.4 (19)	3.5 (9)
Phobic Anxiety	52.4 (13.1)	28.8 (74)	14.8 (38)	14.0 (36)
Paranoid Ideation	45.3 (9.4)	14.4 (37)	8.9 (23)	5.4 (14)
Psychoticism	49.1 (11.5)	17.5 (45)	8.9 (23)	8.6 (22)
**Cognitive Reserve Index Questionnaire (CRIq)**				
CRIq-total score	116.7 (18.3)	–		–
CRIq-education	105.7 (14.9)	–		–
CRIq-working activities	109.4 (18.5)	–		–
CRIq-leisure time	123.8 (19.2)	–		–

*T, t scores.*

### Predictors of Psychological Distress

#### Illness Severity and Demographics

Results revealed a significant association between scores at the Somatization subscale and hospitalization (Estimate = −8.77; SE = 3.11; Standardized Estimate = −0.76; *t* = −2.81; *p* < 0.01), with higher scores in patients who were not hospitalized because of COVID-19. Results also showed significant association between sex and Positive Symptom Total (Estimate = 3.25; SE = 1.58; Standardized Estimate = 0.28; *t* = 2.4; *p* < 0.05) with higher levels of psychological distress in female participants. Similarly, no other illness severity indices were found to be significantly associated with any of the SCL-90-R scores.

#### Subjective Cognitive Difficulties

Results of the linear regression analyses revealed that the COVID-19-related subjective cognitive difficulties represent a predictor of psychological distress, with significant association with the Global Severity Index score (Estimate = −11.43; SE = 1.66; Standardized Estimate = −0.96; *t* = −6.85; *p* < 0.001), Positive Symptom Distress Index (Estimate = −8.00; SE = 1.56; Standardized Estimate = −0.76; *t* = −5.12; *p* < 0.001), and Positive Symptom Total (Estimate = −10.70; SE = 1.63; Standardized Estimate = −0.92; *t* = −6.54; *p* < 0.001). Results also show that the presence of subjective cognitive difficulties was significantly associated to all the nine primary symptoms dimension of the SCL-90-R (for all, *p* < 0.001; see Table 3); in particular, higher scores at the SCL-90-R were found in those patients who experienced cognitive difficulties after being ill with COVID-19. Importantly, no significant relations emerged between SCL-90-R scores and the presence of subjective cognitive difficulties before COVID-19.

**TABLE 3:** Results of the regression analysis showing that SCL-90-R primary symptoms dimensions are predicted by subjective cognitive difficulties.

SCL-90-R	Subjective cognitive difficulties after COVID-19		
Primary symptoms dimensions	Estimate (SE)	Standardized Estimate	*t*
Somatization	−9.19 (1.64)	−0.79	−5.57**
Obsessive–Compulsive	−13.23 (1.71)	−1.05	−7.71**
Interpersonal Sensitivity	−6.71 (1.57)	−0.65	−4.27**
Depression	−11.12 (1.77)	−0.90	−6.26**
Anxiety	−10.21 (1.66)	−0.88	−6.14**
Hostility	−4.37 (1.08)	−0.61	−4.02**
Phobic Anxiety	−8.12 (2.07)	−0.60	−3.92**
Paranoid Ideation	−5.90 (1.38)	−0.65	−4.26**
Psychoticism	−9.73 (1.63)	−0.86	−5.93**

*SE, standard error.*

**p < 0.001.

#### Cognitive Reserve

**FIGURE 1:**
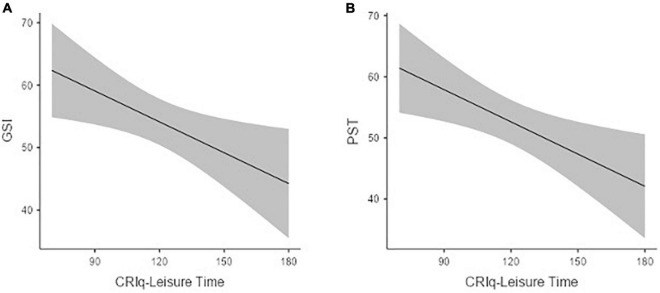
The association between CRIq-leisure time score and the SCL-90. Global severity index **(A)** and positive symptom total **(B)**.

Results revealed the presence of significant negative associations between CRIq-leisure time score and the following SCL-90-R global indices: Global Severity Index (Estimate = −0.16; SE = 0.06; Standardized Estimate = −0.26; *t* = −2.45; *p* < 0.05; see Figure 1A) and Positive Symptom Total (Estimate = −0.17; SE = 0.06; Standardized Estimate = −0.28; *t* = −2.67; *p* < 0.01; see Figure 1B).

Levels of CRIq-leisure time also predicted the following primary symptoms dimension scores: Obsessive–Compulsive (Estimate = −0.18; SE = 0.06; Standardized Estimate = −0.27; *t* = −2.65; *p* < 0.005), Interpersonal Sensitivity (Estimate = −0.14; SE = 0.06; Standardized Estimate = −0.26; *t* = −2.24; *p* < 0.05), Depression (Estimate = −0.16; SE = 0.06; Standardized Estimate = −0.25; *t* = −2.33; *p* < 0.05), Anxiety (Estimate = −0.13; SE = 0.06; Standardized Estimate = −0.21; *t* = −1.96; *p* = 0.05), and Psychoticism (Estimate = −0.15; SE = 0.06; Standardized Estimate = −0.26; *t* = −2.36; *p* < 0.05).

## Discussion

The main goal of this study is to add new knowledge to the growing research field investigating the post-COVID-19 symptoms. More specifically, we aimed to evaluate symptoms of psychological distress in patients who recovered from COVID-19, confirming what the literature has reported, so far, and investigate the role of illness severity, subjective cognitive failures, and CR in their development and manifestation. To reach our goals, we administered the SCL-90-R scale to a sample of 257 patients who recovered from COVID-19, and we evaluated the role of the above-mentioned variables in the score’s variability. Results show that more than 20% of the patients who recovered from COVID-19 who took part in this study showed clinically relevant symptoms of Anxiety, Depression, Obsessive–Compulsive disorder, Phobic Anxiety, and Somatization (it is interesting to note that, before the pandemic, the Italian prevalence of mental diseases was around 7%, De Girolamo et al., 2014).

If compared with the more recent studies statistics obtained from the Italian population during the pandemic (Delmastro and Zamariola, 2020; Rossi et al., 2020), these data suggest the presence of a significant level of psychological distress in patients who recovered from COVID-19, in line with previous reports (Xu et al., 2020; Bonazza et al., 2020; Janiri et al., 2020; Ma et al., 2020; Rabinovitz et al., 2020; Liyanage-Don et al., 2021; Tomasoni et al., 2021). This result, although not suggesting new evidence, is still contributing to defining the psychological outcomes, of the date somehow mixed (Cooke et al., 2020) of individuals who suffered from COVID-19. Future studies would need to further examine the specific aspects of these psychological symptoms. For example, it would be important to investigate to what extent the Phobic Anxiety symptoms and the Obsessive–Compulsive behaviors are related to possible COVID-19 reinfection.

Coherently with the literature (Hodes and Epperson, 2019; see also Yan et al., 2021), results also indicated that, even in this sample of patients, being female represented a risk factor for the development of psychological distress, and, more specifically, to obtain higher scores at the global index Positive Symptoms Total, which represents the number of SCL-90-R items scored above zero (this latter result, however, confirms what the original authors of the scale found through an investigation about factorial invariance across gender (Derogatis and Cleary, 1977).

Results also indicated that, of the different illness-related variables considered in this study, only hospitalization had a significant role in explaining the variability of these symptoms. Specifically, results revealed a protective role of hospitalization on the scores obtained at the SCL-90-R *Somatization* subscale, which, according to Holi (2003), measures symptoms that have a high prevalence in disorders with suggested functional etiology and that reflects distress arising from bodily perceptions. Surprisingly, no other illness severity indices were found to be significantly associated with any of the SCL-90-R scores. We could hypothesize that, regardless of the severity of COVID-19, just being infected represents, in a historical experience as a worldwide pandemic, a strong enough event to develop and manifest psychological malaise. Although based on the first reading, the lack of other associations between illness severity and psychological distress could not seem in line with the current literature, we also believe that this result could be ascribed to the measurements used and the psychological domains explored.

Taken together, these first set of results are coherent with the findings of Mazza et al. (2020), who reported that both being male and being admitted to the hospital represented protective factors against the development of psychological sequelae after COVID-19.

We hypothesized that being hospitalized may have induced individuals to feel more protected, in a safe space, in which potentially fatal consequences of the disease would have been early detected and treated by the hospital staff. Conversely, being infected and at home, could have made people feeling more vulnerable, lonely, and abandoned because of the disease.

The second set of results revealed a significant association between the presence of subjective cognitive difficulties and the level of psychological distress in patients who recovered from COVID-19.

Specifically, this association was quite broad and robust, as it was significant for each of the SCL-90-R subscales, and it was at the same time very specific since it concerned only the cognitive difficulties that patients report to be present after, but not before, their illness of COVID-19. These findings are coherent with the well-established relation between subjective cognitive failures and affective symptoms (for a meta-analysis see Hill et al., 2016; see also Mahoney et al., 1998), which has been also reported in recent studies, investigating the general population’s mental health during the pandemic (Kelly, 2020; Fiorenzato et al., 2021; Nogueira et al., 2021; Santangelo et al., 2021). Adding new knowledge in the research field investigating psychological sequelae of recovered patients with COVID-19, our findings open new questions to be answered with follow-up studies.

Indeed, in absence of a pre-illness evaluation of both cognitive functions and psychological status, the association that emerged in this study does not give us information about the temporal dynamics of this symptom’s association and can be, by now, read as proof of an association between the patient’ distress and the subjective evaluation of cognitive abilities, and associations that were also documented in other clinical conditions (Goebel et al., 2013; Pranckeviciene et al., 2017). Furthermore, it could be that subjective cognitive difficulties can be intended as a proxy rather than a cause of distress. Our data are currently not able to disambiguate this point, which we believe represents an open issue to investigate.

Finally, the third set of results revealed that CR represents a protective factor against the development of psychological distress symptoms in patients who recovered from COVID-19.

Specifically, a higher frequency of cognitively stimulating activities not directly related to education or occupation, such as going to the movies, driving a car, and managing a bank account, predicts lower levels of Depression, Anxiety, and Obsessive–Compulsive symptoms, expressed as clinically significant by more than 20% of the sample, as well as lower scores at the SCL-90-R sub-scales assessing *Interpersonal Sensitivity* and *Psychoticism*. These associations were also reflected by the ones between CR and two of the three SCL-90-R global indices, namely, the *Global Severity Index*, which is the average score of the 90 items of the questionnaire, and the *Positive Symptom Total*, which is the number of items scored above zero (Derogatis, 1992). It is interesting to note that CR related to leisure time activity showed the most significant results in our analyses considering the social restrictions adopted to control the COVID-19 spread. This makes even more sense if we look at some of the results, such as Interpersonal Sensitivity, Depression, and Psychoticism that are somehow implicated in individuals’ social life and abilities.

To the best of our knowledge, this is the first study pointing out the role of CR and, more specifically, of the reserve build through a lifetime of cognitive and socially stimulating activity, not directly linked to the educational context, as a protective factor against the psychological distress symptoms experienced by patients who recovered from COVID-19. Indeed, in a recent study by Yan et al. (2021), psychological distress was positively correlated with education, and negatively correlated with the frequency of contact with colleagues and with psychological resilience.

### Limitations and Future Directions

Some limitations should be noted when considering our results. First, the lack of a psychological and cognitive assessment that preceded the illness, prevented us from evaluating more rigorously the changes in psychological wellbeing and cognitive functions due to COVID-19. Second is the lack of a control group, either of individuals who have never contracted the virus, or of patients who had a different diagnosis in the same historical period. Recognizing these two as the main limitations of our study, we have directly addressed them by asking participants to evaluate their psychological and cognitive wellbeing before the diagnosis of COVID-19, and adopting an analytic approach aimed to investigate the role of specific COVID-19-related variables, which would not have been evaluable in healthy individuals or patients with different clinical conditions. On the other hand, also asking patients directly about their cognitive and psychological wellbeing could represent a bias *per se* since it is not unobjectionable to their meta-cognitive ability. Furthermore, it should be acknowledged that individuals with psychiatric diseases were excluded from the study; however, this information was only collected by a self-report and consulting clinical records without assessing the presence/absence of diseases through specific tools. Finally, and similarly to the previous consideration, the presence/absence of cognitive difficulties was simply asked from participants without a neuropsychological screening. Also, data about cognitive status before the pandemic should be carefully considered, considering the self-report methodology adopted.

Future development of the present work could take into account these aspects by planning multiple follow-ups and testings of patients who recovered from COVID-19. This would allow not only to overcome these limitations, but also to better define the psychological and cognitive aspects of COVID-19.

## Conclusion

To our opinion, the findings of this study represent a set of crucial information for both researchers and clinicians working with patients who recovered from COVID-19, which is currently more than 4 M only in Italy (World Health Organization). Indeed, our results not only represent an important knowledge advancement in the study of the factors associated with psychological distress in patients who recovered from COVID-19, but it emphasizes the importance of a multidisciplinary assessment, where clinical, psychological, and cognitive evaluations should be integrated.

Gaining insight into the dynamic association between these aspects might help not only in identifying individuals who are at increased risk early on, but also in providing adequate support to mitigate mental health problems in patients who recovered from COVID-19.

## Data Availability Statement

The raw data supporting the conclusions of this article will be made available by the authors, without undue reservation.

## Ethics Statement

The studies involving human participants were reviewed and approved by the Ethics Committee of the Padova University Hospital. The patients/participants provided their written informed consent to participate in this study.

## Author Contributions

ED, MD, PI, BV, and DM designed the study. ED analyzed the data and drafted the manuscript. All authors contributed to data collection, interpretation, and manuscript revision.

## Conflict of Interest

The authors declare that the research was conducted in the absence of any commercial or financial relationships that could be construed as a potential conflict of interest.

## Publisher’s Note

All claims expressed in this article are solely those of the authors and do not necessarily represent those of their affiliated organizations, or those of the publisher, the editors and the reviewers. Any product that may be evaluated in this article, or claim that may be made by its manufacturer, is not guaranteed or endorsed by the publisher.

## Footnotes

1 1N = 227.

2 2N = 236.
